# Extracellular matrix abnormalities in Hutchinson–Gilford progeria fibroblasts: a specific defect in collagen IV and basement membrane architecture?

**DOI:** 10.1016/j.mbplus.2026.100202

**Published:** 2026-07-15

**Authors:** Shreya Karmacharya, Arthur Lauri Pasanen-Zentz, Franziska Busse, Nils Michael Kronenberg, Diego Rodrigo Alvarez Chavez, Suzan Al-Gburi, Tristan Lerbs, Bent Brachvogel, Clara Velmans, Iliana Tantcheva-Poor, Raimund Wagener, Mats Paulsson, Carien M. Niessen, Malte Christian Gather, Thomas Krieg, Alvise Schiavinato

**Affiliations:** a Center for Biochemistry, Medical Faculty, University of Cologne, Cologne, Germany.; b Department of Chemistry and Biochemistry, Humboldt Centre for Nano- and Biophotonics, University of Cologne, Cologne, Germany.; c Translational Matrix Biology, Faculty of Medicine, University of Cologne, Cologne, Germany; d Department of Dermatology and Venereology, Medical Faculty and University Hospital of the University of Cologne, Cologne, Germany; e Institut für Allgemeine Pathologie und Pathologische Anatomie, Faculty of Medicine, University of Cologne, Cologne, Germany; f Cologne Excellence Cluster on Cellular Stress Responses in Aging-Associated Diseases (CECAD), University of Cologne, Cologne, Germany; g Department of Pediatrics and Adolescent Medicine, Experimental Neonatology, Faculty of Medicine and University Hospital Cologne, University of Cologne, Cologne, Germany; h Center for Molecular Medicine Cologne (CMMC), University of Cologne, Cologne, Germany; i Institute of Human Genetics, Faculty of Medicine and University Hospital Cologne, University of Cologne, Germany.; j Department Cell Biology of the Skin, Cologne Excellence Cluster on Cellular Stress Responses in Aging Associated Diseases (CECAD), University of Cologne, Cologne, Germany

**Keywords:** Progeria, Basement membrane, Collagen IV

## Abstract

Hutchinson–Gilford progeria (HGPS) is a rare genetic disorder characterized by clinical features that mimic accelerated aging. The classical form of progeria is caused by a heterozygous pathogenic variant in in the *LMNA* gene resulting in the truncated lamin A protein progerin that accumulates in the nuclear envelope and exerts toxic effects on connective tissue and bone growth. Although connective tissue abnormalities are a hallmark of the disease, the extracellular matrix (ECM) produced by HGPS fibroblasts has not been systematically characterized. Here, we combined analysis of four RNA-seq datasets with functional and secretome analyses of primary dermal fibroblasts from an infant with HGPS. Transcriptomic analyses identified enrichment of basement membrane–related pathways and consistent dysregulation of the matrisome gene *COL4A1*. Patient fibroblasts recapitulated canonical HGPS phenotypes, including progerin accumulation, altered nuclear morphology, reduced proliferation, early senescence, and changes in cell mechanics. Secretome proteomics revealed selective downregulation of core basement membrane components, including collagen IV chains with corresponding transcriptional reductions and aberrant collagen IV deposition in vitro. In 3D skin equivalents, HGPS fibroblasts failed to support proper dermo-epidermal basement membrane assembly, a defect mirrored by reduced collagen IV staining in patient skin tissue. Together, these findings identify impaired basement membrane composition and organization as a previously underappreciated feature of HGPS and suggest that ECM remodeling may contribute to cutaneous pathology and potentially broader disease manifestations.

## Highlights

• HGPS fibroblasts show selective dysregulation of core basement membrane components, including collagen IV chains, identified by integrated transcriptomic and secretome proteomics analyses.
• Impaired dermo-epidermal basement membrane assembly in 3D skin equivalents and patient skin tissue identifies basement membrane remodeling as a previously underappreciated feature of Hutchinson–Gilford progeria syndrome.

## Introduction, results and discussion

HGPS is an ultra-rare genetic syndrome with an estimated incidence of 1 case per 4–8 million live births and is classified as a segmental progeroid disorder. Affected individuals exhibit a constellation of multisystemic features including growth deficiency with bone abnormalities, characteristic facies, alopecia, hearing loss, muscular atrophy, progressive joint contractures, subcutaneous lipodystrophy, and sclerotic skin. Without intervention, progressive premature atherosclerosis results in death at age 14.5 years on average [1]. The most common cause of the disease is the recurrent heterozygous de novo variant c.1824C > T in the *LMNA* gene resulting in the production of a truncated form of the intermediate filament lamin A [2]. Accumulation of this truncated protein, termed progerin, causes the characteristic morphological changes of the nuclear envelope [3]. These structural abnormalities are associated with a constellation of cellular alterations, such as telomere attrition, DNA repair deficiencies, dysregulated gene expression, aberrant epigenetic regulation, and genomic instability inducing premature senescence. However, the consequences of these mutations and how these cellular defects are translated into the pleiotropic clinical manifestations of the syndrome are still insufficiently investigated. Interestingly, the clinical phenotype and evidence from pathological examinations and studies in mouse models revealed perturbations in connective tissue, with the skin being one of the most consistently affected [4], [5], [6], [7], [8], [9], [10], [11], [12] associated with profound dysregulation of extracellular matrix (ECM) components [13]. Based on these observations, it has been argued that HGPS represents a form of connective tissue disorder [4], [14], [15]. However, despite this apparent association, the ECM produced by HGPS fibroblasts has never been systematically investigated.

**Fig. 1:**
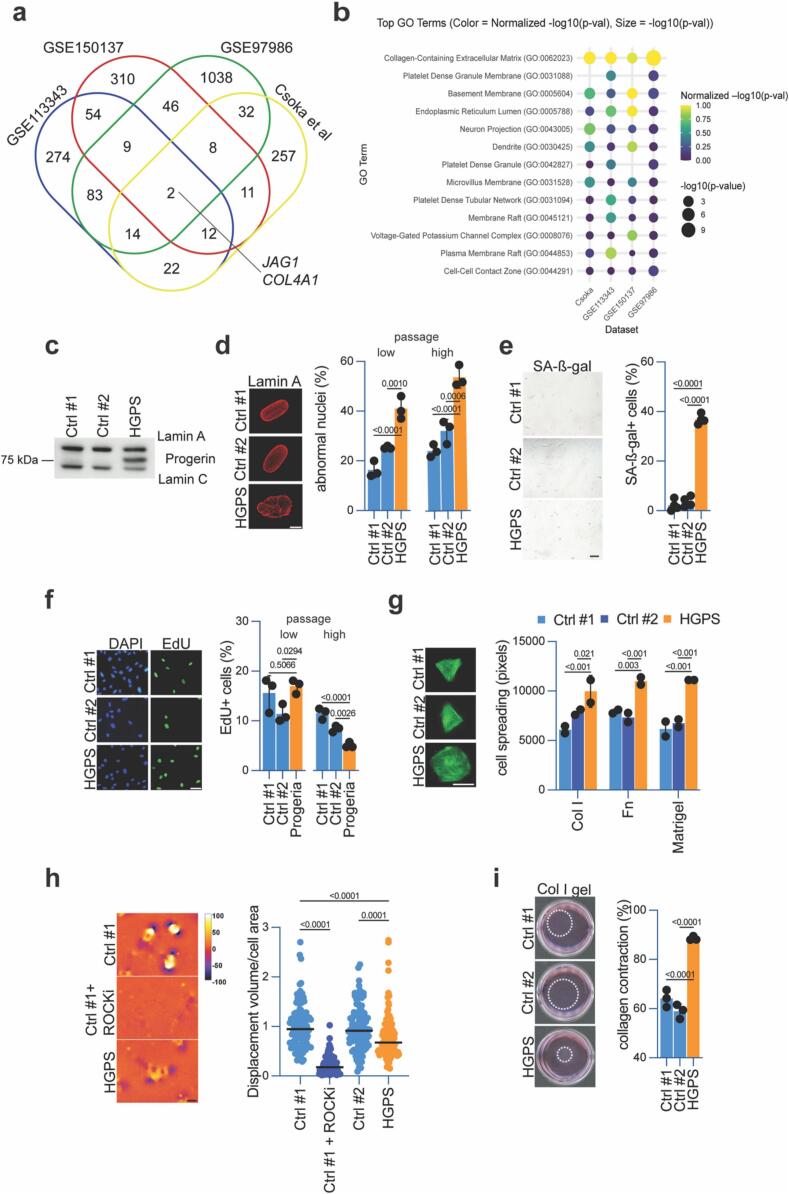
**Characterization of the patient dermal fibroblasts.** (**a**) Venn diagram of differentially expressed genes showing that only two genes, *COL4A1* and *JAG1* are commonly dysregulated (log2 fold change ≤ − 1 or ≥ 1, *p* < 0.05) in the transcriptomic profiles of HGPS fibroblasts across four previously published datasets. (**b**) Balloon plot of the gene ontology for the cell component aspect showing enrichment of the collagen containing ECM and basement membrane terms across the different datasets. (**c**) Immunoblot for lamin A/C from cell lysates of control and the HGPS patient dermal fibroblasts. The mutated progerin product is clearly visible only in the patient. (**d**) Representative images and quantification of nuclei with abnormal morphology as revealed by lamin A/C immunofluorescence in the indicated fibroblasts at early and later passages. Each data point represents the analysis of at least 100 cells. *N* = 3 Scale bar: 5 μm (**e**) Increased expression of SA-β-Gal by early passage HGPS compared to the control fibroblasts. *N* = 4, 100 cells per data point. Scale bar: 100 μm (**f**) Representative images and quantification of EdU incorporation rates in the indicated fibroblasts at early and later passages. N = 3, >100 cells per sample. Scale bar: 50 μm (**g**) Increased area of HGPS fibroblasts after 2 h spreading on the indicated ECM substrates. N = 3, at least 20 cells were measured for each data point. Scale bar: 50 μm (**h**) Representative ERISM displacement maps of control and HGPS fibroblasts plated on a fibronectin-coated stress sensor. Scale bar: 50 μm. The graph on the right shows the aggregated results of 4 independent experiments normalized to control #1. Note the complete loss of displacement in cells treated with the ROCK inhibitor Y-27632 (10 μM), which served as a positive control, and the significant reduction in displacement in HGPS cells when compared to both control lines. (**i**) Representative images and quantification of collagen I gel contraction after 3 h from embedding the cells. HGPS fibroblasts were faster at contracting the gels. *N* = 3. One-way ANOVA was used for statistical comparisons in all panels except panel (e), which was analyzed by two-way ANOVA.

**Fig. 2:**
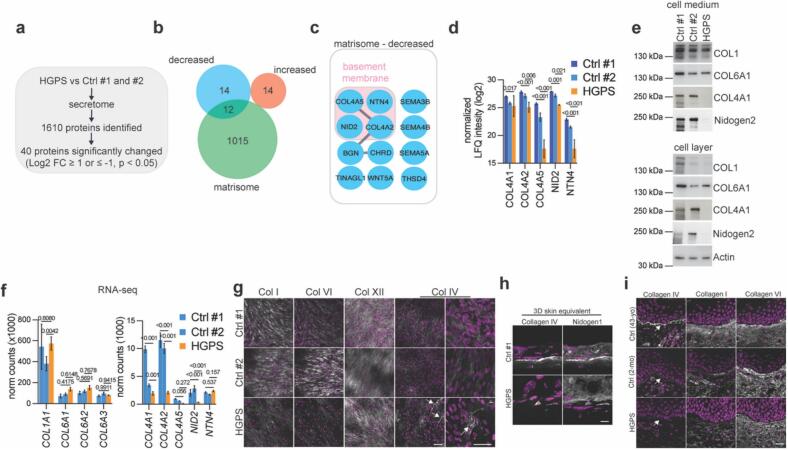
**Characterization of the ECM produced by control and HGPS dermal fibroblasts.** (**a**) Schematic overview of the experimental workflow for the secretome analysis. HGPS and control fibroblasts were kept confluent and treated with ascorbate for one week and then shifted to serum-free medium with ascorbate for 72 h. Conditioned media were then collected, digested and subjected to label-free quantitative mass spectrometric analysis. (**b**) Venn diagram of proteins significantly decreased and increased in HGPS fibroblasts and their distribution within the human matrisome dataset. (**c**) Physical STRING network of the 12 matrisome proteins decreased in the secretome of HGPS fibroblasts. Basement membrane components are highlighted. (**d**) Label-free mass spectrometry quantification of the indicated proteins in the secretome of control and HGPS fibroblasts. N = 3. Two-way ANOVA was used for statistical comparisons. (**e**) Immunoblots for the indicated ECM components in the cell layers and conditioned media of control and HGPS fibroblasts. (**f**) RNA sequencing revealed reduced expression of collagen IV chains and nidogen-2 in HGPS fibroblasts, whereas other collagens were not affected. (**g**) Collagens of control and HGPS fibroblasts were labeled with the indicated antibodies. A different fibril architecture was evident only for collagen IV (arrows). Scale bars: 100 μm (**h**) Skin equivalents generated using HaCaT cells and control or HGPS fibroblasts embedded in collagen I were stained for collagen IV and nidogen 1. A basement membrane-like structure was visible only in the skin equivalent generated with the control fibroblasts. Scale bar: 15 μm (**i**) Skin sections of two healthy control donors and the HGPS proband were stained with the indicated collagen antibodies. Despite comparable collagen I and VI labeling, the collagen IV labeling of the basement membrane at the dermo-epidermal junction (arrows) was weaker in the patient. Scale bar: 20 μm.

To better understand the molecular basis of this association, we analyzed four available transcriptomics datasets of HGPS fibroblasts [16], [17], [18], [19] and identified the basement membrane component *COL4A1* as the only commonly dysregulated matrisome encoding gene (Fig. 1a). Moreover, gene ontology analysis revealed consistent enrichment of the terms “collagen-containing extracellular matrix” and “basement membrane” (Fig. 1b). To obtain independent validation, we characterized dermal fibroblasts from a 3-month-old male infant with HGPS carrying the classical recurrent heterozygous de novo variant c.1824C > T, who was treated at our clinic, alongside two control fibroblast cultures from age-matched healthy donors. Western blot analysis confirmed the accumulation of progerin in the patient cells (Fig. 1c). Next, we assessed classical progeria-associated cellular phenotypes, including altered nuclear morphology, reduced proliferation, and increased expression of senescence-associated β-galactosidase (SA-β-Gal). Notably, SA-β-Gal expression was elevated even at early passages in patient fibroblasts, while decreased proliferation and nuclear abnormalities became more evident at later passages (Fig. 1d-f), in line with previous findings [3], [20]. Given that progerin-induced nuclear envelope defects can impact actin cytoskeleton dynamics [21], [22], we employed cell spreading and traction and gel contraction assays. HGPS fibroblasts exhibited increased cell spreading on collagen I, fibronectin, and Matrigel after two hours (Fig. 1g). Elastic Resonator Interference Stress Microscopy (ERISM) [23], [24] revealed reduced force generation by HGPS fibroblasts cultured on fibronectin-coated 2D sensors (Fig. 1h), whereas in a 3D collagen I gel contraction assay, patient fibroblasts contracted the matrix more rapidly than controls (Fig. 1i). The reduced ERISM traction on 2D substrates, yet faster 3D lattice contraction by HGPS fibroblasts, likely reflects context-dependent cell–matrix coupling rather than a global change in contractility. Together, these results confirm that the patient fibroblasts recapitulate cellular phenotypes of HGPS. To further investigate if progerin expression affects extracellular ECM composition, we cultured control and patient fibroblasts with ascorbate for one week, followed by a 48-h incubation in serum-free medium supplemented with ascorbate. The conditioned media were analyzed via label-free proteomics, enabling the identification and quantification of 1610 proteins (Supplementary Table 1). Of these, 26 proteins were significantly decreased and 14 increased in abundance (fold change ≤ − 1 or ≥ 1, adjusted *p*-value <0.05) in the patient fibroblasts compared to both controls (Fig. 2a). Intersecting these proteins with the human matrisome dataset identified 12 downregulated and no upregulated matrisome components (Fig. 2b). Intriguingly, among the 12 downregulated components, four belong to the core matrisome (COL4A5, COL4A2, NTN4, and NID2), all of which are key constituents of the basement membrane (Fig. 2c-d). Immunoblots confirmed selective downregulation of collagen IV and nidogen-2 in patient fibroblasts (Fig. 2e) and RNA-seq showed reduced expression for collagen IV and nidogen-2 genes but not for other collagens (Fig. 2f and Supplementary Table 2). This specific disruption of basement membrane components prompted further investigation. Fibroblasts cultured on glass coverslips for one week with ascorbate were stained for core matrisome components. Collagen I, VI, and XII appeared similarly organized in both patient and control samples, although collagen I and VI were sparser in the patient cells. In contrast, collagen IV (detected via an antibody directed against the α1 chain) specifically showed aberrant deposition in progeria fibroblasts, indicating a selective defect in this basement membrane component (Fig. 2g).

To explore whether HGPS fibroblasts form an altered dermo-epidermal basement membrane in a tissue-like environment, we generated 3D skin equivalents using collagen I, patient or control fibroblasts, and HaCaT keratinocytes. While the control fibroblasts supported the formation of a nascent basement membrane, as evident by linear collagen IV and nidogen-1 staining between the fibroblast and keratinocyte layers, this structure was absent in the skin equivalents containing patient fibroblasts (Fig. 2h). Notably, fibroblasts are the principal source of collagen IV during dermo-epidermal basement membrane assembly in organotypic skin cultures [25], [26].

Finally, immunostaining of skin sections from the patient and two healthy controls revealed weaker collagen IV staining in the dermo-epidermal zone in the patient sample, but not around blood vessels (not shown), while collagen I and VI appeared comparable (Fig. 2i). Collectively, these data suggest that dermo-epidermal basement membrane assembly is specifically impaired in progeria. At present the exact molecular mechanism how progerin affects the regulation of ECM arrangement remains unclear. ECM abnormalities in a progeria mouse model have been attributed to defective Wnt signaling [12]. The identification of *JAG1* as the other gene commonly dysregulated in progeria fibroblasts suggests a further link with Notch signaling [27], a pathway that was already shown to regulate collagen IV gene expression in fibroblasts [28]. Reorganization of lamina-associated chromatin domains and reshaping of the epigenetic landscape could also directly affect transcription of basement membrane genes in HGPS fibroblasts [16]. Progerin also perturbs nucleo-cytoskeletal coupling and actin dynamics [21], [22]; the altered spreading, traction and gel-contraction behaviour we observed in the patient fibroblasts confirms changes in cell mechanics and mechanotransduction that are known to feed back on matrix gene expression and assembly [29]. Moreover, progerin expression induces endoplasmic reticulum stress and impairs protein quality-control pathways [27], [30], which could affect secreted proteins such as those involved in basement membrane assembly. Our study has limitations. The functional and secretome analyses rely on fibroblasts from a single HGPS donor compared with two control lines and will need to be confirmed in additional patient-derived lines. We also cannot fully exclude that part of the collagen IV changes reflects a secondary effect of cellular senescence rather than a mechanism strictly specific to progerin accumulation. However, our observations indicate that quantitative and qualitative remodeling of the ECM, and specifically the basement membrane, may be part of the complex phenotype of HGPS. It remains to be determined whether these observed alterations contribute to the well-known sclerodermoid features of the skin in these patients or are responsible for more specific symptoms, such as fragility, dyspigmentation and nail dystrophy. Although the underlying molecular mechanism leading to the ECM alteration remains to be elucidated, it is interesting to note that basement membrane remodeling also occurs during normal aging suggesting the involvement of a shared mechanism [31], [32]. A decline of collagen IV at the dermo-epidermal junction is, for instance, a recognized feature of aged human skin [33].

## Material and methods

### Cell culture

Patient fibroblasts were established by outgrowth from skin biopsy as previously described [34]. Neonatal foreskin fibroblasts from Invitrogen and ATCC (HFF-1, SCRC-1041) were used as control line #1 and #2, respectively. Fibroblasts were cultured in DMEM/F12 supplemented with 10% FBS and 1% penicillin/streptomycin and controlled for mycoplasma contamination. Primary fibroblasts were used from passage 5 to passage 20. For ECM production cells were kept in complete medium supplemented with ascorbate for 7 days. Medium was changed every other day.

### Mass spectrometry analysis

Secretome analysis was performed on conditioned media collected from confluent cultures. Briefly, 1 × 10^5^ cells per well (6-well format) were grown to confluency with ascorbate supplementation, after which the medium was replaced with serum-free medium containing ascorbate for an additional three days. Ascorbate was added every other day. The medium was collected, acetone precipitated, in-solution digested and loaded onto SDB-RP StageTips according to facility protocols. LC-MS/MS analysis was carried out at the CECAD proteomics facility (Cologne) using a Q Exactive Plus Orbitrap instrument interfaced with an EASY nLC system (Thermo Fisher Scientific). Chromatographic separation was performed on an in-house packed C18 column (50 cm × 75 μm i.d., 2.7 μm Poroshell EC120, Agilent) at 250 nL/min. Mobile phase A consisted of 0.1% formic acid in water; mobile phase B of 0.1% formic acid in 80% acetonitrile. The following gradient was applied: 4–6% B over 5 min, 6–23% B over 120 min, 20–54% B over 7 min, 54–85% B over 6 min, followed by column wash and re-equilibration. Data were acquired in data-dependent mode. Full MS1 scans spanned 300–1750 *m*/*z* at 70,000 resolution. The ten most intense precursors were selected for HCD fragmentation (normalized collision energy 27%, isolation window 2.1 Th); fragment ions were recorded in the Orbitrap at 17,500 resolution. The AGC target was 5 × 10^5^ with a maximum injection time of 60 ms, and dynamic exclusion was set to 25 s. Raw files were processed in MaxQuant (v1.5.3.8) against the canonical UniProt human proteome (UP5640, accessed 20 January 2022), with default settings and match-between-runs enabled across replicates. Downstream statistical analysis was performed in Perseus (v1.6.15). The dataset was filtered to remove reverse hits, known contaminants, and peptide-only identifications. After applying completeness filters within replicate groups, missing LFQ intensity values were imputed by sigma downshift using default parameters. Group comparisons were carried out using FDR-controlled Student's *t*-tests (s0 = 0.2).

### RNA-sequencing and analysis

Total RNA was extracted with TRIzol™ (Invitrogen). RNA-sequencing was performed by Novogene Europe using 150-bp paired-end sequencing on an Illumina platform. After cDNA library construction, sequencing, and filtering, reads were mapped to the *Homo sapiens* reference genome (GRCh38.p14) using Hisat2 v2.0.5. Read numbers mapping to each gene were obtained using FeatureCounts v1.5.0-p3 and converted to Fragments Per Kilobase of transcript per Million mapped reads (FPKM). Differential gene expression analysis was done using DESeq2 in R. RNA-seq unnormalized counts from previously published datasets were downloaded from GEO (GSE150137, GSE113343, GSE97986) and used for differential gene expression analysis using DESeq2 in R after pre-filtering the count matrices for transcripts with a count greater than one. Each dataset was processed and normalized independently in DESeq2 using its default median-of-ratios size-factor normalization, and significantly differentially expressed genes were called separately for each dataset (|log2 fold change| ≥ 1). To avoid cross-platform and batch effects, the datasets were not merged, but rather analyzed separately. The intersection of significantly dysregulated matrisome genes among the lists was used for Fig. 1a.

### Immunofluorescence and microscopy

For immunofluorescence, cells were grown on 12 mm glass coverslips in 24-well plates for 24–48 h and fixed with 4% PFA/PBS for 15 min. To visualize ECM networks, 5 × 10^4^ cells were seeded on coverslips, cultured for 7 days with ascorbate-containing medium refreshed every second day, and fixed with 4% PFA/PBS or ice-cold methanol/acetone (−20 °C, 10 min). Samples were permeabilized with 0.5% NP-40/PBS for 10 min and blocked with 1% FBS/PBS for 30 min. Primary antibody incubations were carried out for 1 h at RT using antibodies against collagen I (Millipore AB758), collagen IV (Chemicon AB769), collagen VI [35], collagen XII (kind gift of Dr. Manuel Koch), nidogen 1 and nidogen 2 [36]. Highly cross-adsorbed secondary antibodies conjugated to AlexaFluor 488, 555, or 647 (Thermo Fisher Scientific; 1:400 in 1% FBS/PBS) and DAPI (0.1 μg/ml; Sigma-Aldrich) were applied for 1 h at RT. Fluorescence images were acquired on a Leica Stellaris 8 confocal microscope. For tissue staining, paraffin-embedded skin sections from the patient and two healthy controls (aged 43 years and 2 months) were incubated overnight with primary antibodies without prior antigen retrieval, and secondary antibodies were applied the following day as described above.

### Cell lysis and western blotting

Cells were plated in 6-well plates and allowed to grow for at least 48 h. To collect conditioned media for ECM protein analysis, the culture medium was replaced with serum-free medium for 72 h, with ascorbate supplemented every two days. Cell lysates were prepared in 4% SDS/PBS, followed by sonication and centrifugation, and protein concentration was determined by BCA assay. Actin served as a loading control.

### EdU proliferation assay

Proliferating cells were labeled with the thymidine analogue EdU. Briefly, 1 × 10^4^ cells were seeded on glass coverslips and grown for 24 h, then pulsed with 10 μM EdU for 1 h. Following fixation with 4% PFA/PBS, EdU incorporation was detected using the Cell Proliferation Kit III (PromoKine) according to the manufacturer's protocol.

### SA-β-Gal assay

Senescence-associated β-galactosidase activity was detected using the Senescence β-Galactosidase Staining Kit (Cell Signaling) per the manufacturer's instructions. Cells (1 × 10^4^) were seeded in 24-well plates and cultured for 2–4 days before fixation with 4% PFA/PBS. Each well received 250 μl of freshly prepared β-galactosidase staining solution (pH 6.0), and plates were incubated overnight at 37 °C without CO₂. Nuclei were counterstained with DAPI before transferring cells to 70% glycerol for counting.

### Cell spreading

24-well plates were coated overnight at 4 °C with either collagen I (30 μg/ml; Corning), fibronectin (10 μg/ml; Sigma), or Matrigel (10 mg/ml; Corning), and subsequently blocked with 1% BSA in PBS for 2 h at room temperature. A total of 2.5 × 10^3^ fibroblasts were seeded per well and allowed to adhere and spread for 30, 60, or 120 min. Cells were then fixed in 4% PFA and stained with AlexaFluor-488 phalloidin (Cell Signaling). Images were acquired on a Zeiss Axiophot fluorescence microscope and cell spreading area was quantified using ImageJ.

### Gel contraction assay

Collagen lattices were prepared as previously described [37]. Briefly, a neutralized collagen mixture was prepared on ice by combining 0.92 ml of 1.76× DMEM, 0.20 ml collagen (3 mg/ml in 0.2% acetic acid), 0.04 ml of 0.1 M NaOH, 0.19 ml FCS, and 0.67 ml of a fibroblast suspension. Each lattice contained 2.5 × 10^3^ cells in a total volume of 2 ml and was poured into 3 cm bacteriological-grade dishes. Polymerization was allowed to proceed at 37 °C, and lattice contraction was assessed 3 h after casting by scanning the dishes against a black background using a digital scanner.

### Organotypic culture of 3D skin equivalent

Organotypic raft cultures were generated as described previously with minor modifications [38]. Briefly, control and HGPS fibroblasts (4 × 10^5^ per raft) were embedded in rat tail collagen (final concentration 4 mg/ml) and allowed to polymerize in 12-well plates at 37 °C. Collagen plugs were overlaid with fibroblast medium and cultured for 24–72 h. HaCaT keratinocytes (6 × 10^5^ per raft) were then seeded onto the collagen plugs. After two days, rafts were lifted to the air–liquid interface on sterile metal grids and cultured with ascorbate and medium changes every two days. Rafts were harvested 7 days after air lift for fixation, embedding and sectioning.

### ERISM measurements

Elastic Resonator Interference Stress Microscopy (ERISM) was performed using a custom-built setup similar to Kronenberg et al. [23]. Monochromatic light from a tunable white-light source was directed into an inverted microscope (Eclipse Ti, Nikon) to provide collimated epi-illumination of the ERISM stress sensor that serves as substrate for the investigated cells. The sensor comprised an optical microcavity composed of an ∼8 μm thick film of a high-index elastomer layer (QGel920, CHT) deposited on a Ta₂O₅-coated glass substrate [24]. The interference pattern generated within the cavity upon monochromatic illumination was imaged with a sCMOS camera (Zyla 5.5, Andor) while the illumination wavelength was scanned from 550 to 750 nm in 1 nm steps, yielding a reflectance spectrum for each pixel. Phase-contrast images of the cells were automatically acquired after each wavelength scan.

For measurements, a microcavity with a stiffness of ∼19 kPa was coated with fibronectin (10 μg/ml) for 3 h and a 2 × 2 silicon chamber (Ibidi) was placed onto the cavity. Approximately 750 cells were seeded per chamber (progeria fibroblasts in chamber 1; controls in chambers 2–4). After 24 h of incubation at 37 °C, cells in chamber 3 were treated with Y-27632 (10 μM) for 1 h before imaging all chambers. All measurements were performed under physiological conditions in an on-stage incubator. Microcavity thickness maps were generated using a custom Python code that compares experimental reflectance to transfer-matrix simulations. Displacement maps were obtained after background subtraction in ImageJ; deformations >15 nm (or > 10 nm for Y-27632) were used for analysis. Mean displacement, displacement area, and cell area were manually quantified in ImageJ and normalized to control #1. Indentation volume was defined as mean displacement × displacement area, and corrected mean displacement as indentation volume normalized to cell area.

## Declaration of generative AI and AI-assisted technologies in the manuscript preparation process

During the preparation of this work, the author(s) used Claude AI for text editing and proofreading. The author(s) reviewed and edited the output as needed and take full responsibility for the content of the published article.

## CRediT authorship contribution statement

**Shreya Karmacharya:** Writing – review & editing, Investigation. **Arthur Lauri Pasanen-Zentz:** Writing – review & editing, Investigation. **Franziska Busse:** Writing – review & editing, Methodology, Investigation. **Nils Michael Kronenberg:** Writing – review & editing, Methodology. **Diego Rodrigo Alvarez Chavez:** Investigation, Data curation. **Suzan Al-Gburi:** Writing – review & editing, Resources. **Tristan Lerbs:** Writing – review & editing, Resources. **Bent Brachvogel:** Writing – review & editing, Funding acquisition. **Clara Velmans:** Investigation. **Iliana Tantcheva-Poor:** Writing – review & editing, Resources. **Raimund Wagener:** Writing – review & editing, Resources, Funding acquisition. **Mats Paulsson:** Writing – review & editing. **Carien M. Niessen:** Writing – review & editing, Methodology. **Malte Christian Gather:** Writing – review & editing, Supervision, Methodology, Funding acquisition. **Thomas Krieg:** Writing – review & editing, Resources. **Alvise Schiavinato:** Writing – review & editing, Writing – original draft, Investigation, Funding acquisition, Conceptualization.

## Declaration of competing interest

Alvise Schiavinato reports financial support was provided by German Research Foundation. All other authors declare no conflict of interests.

## Data Availability

All data supporting the findings of this study (including proteomics and transcriptome data) are available as supplementary material and upon reasonable request from the corresponding author.

## Appendix A Supplementary data

Supplementary material1: Secretome dataset HGPS and control fibroblasts

Supplementary material2: RNA-sequencing dataset HGPS and control fibroblasts
